# A Smartphone App (WExercise) to Promote Physical Activity Among Cancer Survivors: Randomized Controlled Trial

**DOI:** 10.2196/75839

**Published:** 2025-10-03

**Authors:** Denise Shuk Ting Cheung, Tiffany Wan Han Kwok, Sam Liu, Ryan E Rhodes, Pui Hing Chau, Chi-Leung Chiang, Anne Wing-Mui Lee, Chia-Chin Lin

**Affiliations:** 1 School of Nursing, Li Ka Shing Faculty of Medicine, University of Hong Kong, Academic Building, 3 Sassoon Road, Hong Kong, Hong Kong, 852 39176673; 2 School of Exercise Science, Physical and Health Education, University of Victoria, Victoria, BC, Canada; 3 Center of Clinical Oncology, University of Hong Kong - Shenzhen Hospital, Shenzhen, China

**Keywords:** cancer survivor, M-PAC, mHealth, physical activity, behavior change

## Abstract

**Background:**

Cancer survivors face unique health challenges that may be addressed through physical activity (PA) interventions. Technology-based tools provide innovative, resource-efficient alternatives to traditional approaches, delivering PA interventions.

**Objective:**

This study aimed to examine the effectiveness of a smartphone app (WExercise) in promoting PA among cancer survivors.

**Methods:**

This study was an assessor-blind, 2-arm randomized controlled trial. The intervention group used WExercise, which consisted of automated weekly lessons developed based on the multi-process action control (M-PAC) framework. The control group received written PA recommendations. Ninety-eight physically inactive cancer survivors who had completed curative treatment were recruited from an oncology clinic and the community. Outcomes included exercise behavior (primary), exercise capacity, quality of life, and M-PAC constructs.

**Results:**

The majority (81/98, 82.7%) of participants remained in the study. The proportion of participants completing at least 75% of lessons was 69.44%. For exercise behavior, mixed findings were identified: the intervention group had a significantly greater increase in self-reported moderate-to-vigorous PA compared to the control group at postintervention (mean difference in change 89.02 minutes, 95% CI 34.87-143.16) and 3 months postintervention (mean difference in change 49.37 minutes, 95% CI 8.63-90.10; group × time interaction; *P*=.003), while no significant effect on ActiGraph-measured moderate-to-vigorous PA was observed at postintervention (mean difference in change –8.54 minutes, 95% CI –36.19 to 19.11) and 3 months postintervention (mean difference in change 2.56 minutes, 95% CI –27.29 to 32.41; group × time interaction; *P*=.74). WExercise was also effective in increasing cancer survivors’ exercise capacity but not their quality of life or M-PAC constructs.

**Conclusion:**

WExercise demonstrated a significant effect on increasing self-reported PA, but this was not corroborated with ActiGraph-measured PA. The application may be a useful addition for clinicians aiming to promote physical activity in people with cancer.

## Introduction

Cancer survivors encounter distinct health challenges. Physical activity (PA) has emerged as a safe and recommended intervention to address these challenges [1]. PA has demonstrated significant physical and mental benefits, including reducing anxiety, depression, and fatigue; enhancing quality of life (QoL) and physical function; and lowering the risks of cancer-specific and all-cause mortality [1,2]. Despite the advantages of PA for cancer survivors and established clinical guidelines from the American College of Sports Medicine and the World Health Organization (WHO) [1], PA levels remain suboptimal in this population. A systematic review revealed that only 34.2% of cancer survivors met these guidelines [3], while the National Cancer Institute reported that 36.7% of adult cancer survivors engaged in no PA in 2022, based on the National Health Interview Survey [4].

To promote PA among cancer survivors, technology-based interventions offer novel alternatives to traditional approaches, with benefits such as scalability and resource-efficient implementation. The technology-based PA promotion interventions examined in cancer survivors were categorized as mobile-based interventions (eg, wearable devices and mobile phone apps), web-based interventions (eg, self-monitoring platforms and workshops), and those involving a combination of both mobile-based and web-based interventions [5]. Such approaches can support healthy lifestyle changes by providing real-time feedback, automated reminders, goal setting, and motivational support to maintain exercise routines during cancer survivorship. Systematic reviews and meta-analyses have demonstrated both the feasibility [6] and the effectiveness of these interventions in improving PA outcomes in cancer survivors [5,7,8]. However, researchers often highlight a critical need for high-quality randomized controlled trials (RCTs) that incorporate theory-based designs [9], objective PA measurements [5], and long-term follow-up time points [5,8].

The most frequently used theories in PA promotion trials in cancer survivors were social cognitive theory (building on attitudes and self-efficacy to foster intentions as a means to affect behavior) and the transtheoretical model (initiating healthy behavior changes through a series of progressive stages), which have shown promise in enhancing both PA and dietary habits among cancer survivors [10-12]. However, researchers found that the intention-to-behavior gap (the failure to translate intentions into action) has been prominent in PA-related studies in cancer survivors, underscoring the need for interventions targeting the determinants of both PA intention formation and translation, which may be most effective in promoting PA in cancer survivors [13-15].

The multi-process action control (M-PAC) framework builds on traditional behavior change theories and extends beyond them by focusing not only on determinants of PA intention formation, but also on constructs that bridge the intention-to-behavior gap [16]. The M-PAC framework is a layered approach to behavior change in which reflective processes (eg, instrumental attitude, perceived capability, and perceived opportunity), regulation processes (eg, action and coping planning, self-monitoring, and social support), and reflexive processes (eg, habit and identity formation) build upon each other. The M-PAC framework has been widely applied to promote PA, such as face-to-face programs for the general public [17], self-guided web-based programs for general adults [18,19], telephone counseling programs for cancer survivors [20], and programs for youth with disabilities [21]. However, no RCT has evaluated a smartphone app–based intervention grounded in this framework. In addition to measuring an intervention’s effectiveness in improving PA, it is essential to include exercise capacity and QoL as secondary outcomes, as these are health parameters closely associated with PA in cancer survivors [22]. Furthermore, assessing how an intervention influences the key M-PAC constructs may enhance our understanding of its mechanisms of action.

In view of this research gap, a smartphone app named WExercise was developed on the basis of M-PAC to promote PA among cancer survivors. It comprised weekly lessons delivered primarily through text, images, and video-based content aimed at promoting PA. The app underwent a rigorous iterative development process and was tested for usability [23]. This RCT was designed to examine the effectiveness of the WExercise app on increasing PA (primary outcome), exercise capacity, cancer-specific QoL, and key M-PAC constructs (secondary outcomes) among cancer survivors at postintervention (T1) and 12 weeks post intervention (T2) compared to self-directed PA education.

## Methods

### Study Design

This was an assessor-blinded 2-arm RCT comparing WExercise against self-directed PA education, conducted between November 1, 2022, and May 31, 2024. Reporting in this study followed the CONSORT (Consolidated Standards of Reporting Trials) guidelines.

### Participants

Participants were eligible if they were ≥18 years of age, ≥12 months postcompletion of primary treatment with curative intent, had no metastasis nor recurrence, were not meeting the recommended PA guideline (<150 minutes of moderate-intensity aerobic exercise or <75 minutes of vigorous aerobic exercise per week), had access to a smart device, could read Chinese and communicate in Cantonese or Putonghua, and were cleared by a nurse for unsupervised exercise using a risk screening tool [24]. Those who had a psychiatric disorder, a significant cognitive impairment, or a history of more than one cancer were excluded.

Sample size calculation was based on a previous study of an M-PAC–based exercise telephone counseling intervention for hematologic cancer survivors [20]. The between-group effect size for the increase in moderate-to-vigorous physical activity (MVPA) postintervention was 0.91 (95% CI 0.33-1.48). A more conservative effect size, that is, a moderate-to-large effect of 0.65, was adopted. A sample size of 39 participants per group could provide 80% power to reject the null hypothesis with a significance level of .05. Assuming an attrition rate of 20%, a total of 98 participants was required for this study.

### Procedures

Recruitment took place in the outpatient clinic of a major public hospital in Hong Kong and in community support groups. Potential participants were given a brochure about the study and screened for eligibility before providing written informed consent. After providing written consent, participants were randomly assigned to the WExercise or control group (1:1). Randomization was performed using block randomization with a random block size of 4 or 6. An independent statistician generated the randomization list using a computer and kept a secure copy of the randomization code assignments. The group assignment was sealed in sequentially numbered opaque envelopes. The research assistants responsible for data collection and entering the data were blinded to the group allocation.

### Control Group

Participants of both groups were provided to increase their aerobic exercise levels to 150 minutes of moderate aerobic activity or 75 minutes of vigorous aerobic exercise per week. Participants in the control group received a 1-page written information sheet regarding PA for cancer survivors [1] and exercise safety precautions extracted from the Center for Health Protection of the Hong Kong Government [25]. After providing the information sheet, a trained research assistant answered any questions that the participants had.

### Intervention Group

Participants in the intervention group were additionally provided with individualized access to the WExercise app, which had 12 lessons, including 10 automated weekly lessons aimed at promoting PA and 2 additional lessons on introduction and safety precautions in the first week. The app, developed using an app design platform (Pathverse [26]), contained a combination of text, educational videos, infographics, and special features, including integrating data from cellphones (eg, step count) for self-monitoring, quizzes to strengthen participation, and a diary for logging PA sessions to reflect their progress. Each lesson takes around 20‐30 minutes to complete, depending on individual progress. Participants downloaded the app with the assistance of trained research assistants and were provided with a brief written manual introducing the app features.

The weekly lessons were designed to develop reflective, regulatory, and reflexive processes of cancer survivors to achieve a behavioral pattern of action control to engage in PA, based on the M-PAC framework and the adoption of different behavior change techniques. The structure of lessons was as follows: (1) lessons 1-3: initiating and ongoing reflective processes (instrumental attitude, perceived capability, affective attitude, and perceived opportunity), which are the key to forming intention and beginning the translation of intention into behavior; (2) lessons 4-8: behavioral regulation through action and coping planning, self-monitoring, self-regulating alternative activities, emotion regulation, and building a supportive social and physical environment; and (3) lessons 9-10: forming habits and identity (reflexive processes) for sustaining action control [1]. Participants were sent reminders (3 days apart) by instant messaging each time they had a lesson. The app was tested for usability, and details of the intervention have been described elsewhere [23].

### Safety Precautions

At study entry, participants were screened by a trained nurse for contraindications to exercise based on a risk screening tool covering hematologic, musculoskeletal, systemic, gastrointestinal, cardiovascular, pulmonary, and neurologic conditions [24]. Participants of both groups received written information about safety precautions during exercise, for example, signs and symptoms of exercise intolerance and actions to take in the event of adverse events such as musculoskeletal injury or dizziness (ie, stop exercising and consult physicians). Any adverse events were reported to research personnel and documented.

### Data Collection

Data were collected face-to-face by trained research assistants on university campuses for both groups at baseline (T0), postintervention (10 weeks later; T1), and 12 weeks postintervention (22 weeks later; T2).

#### Primary Outcome

PA behavior was operationalized as time spent in MVPA measured in both objective and self-reported ways. For objective measurement, participants were asked to wear an ActiGraph GT3X (an accelerometer that measures intensity-specific PA) on their nondominant wrist for 7 consecutive days at T0, T1, and T2. Data were collected using a 30 Hz frequency, converted to 60-second epochs, and processed using ActiLife 6.13.4 software (ActiGraph LLC). The criterion for data inclusion was at least 10 hours of wear time on at least 3 days [27]. Nonwear time was defined as intervals of at least 60 minutes of consecutive zero counts with allowance for 1‐2 minutes of interruption. Average time (minutes) per day spent in MVPA (≥2420 counts per minute [28]) was recorded. The self-reported measure was the Godin Leisure Time Exercise Questionnaire (GLTEQ), which contains 6 items to assess the average frequency and duration of aerobic exercise in the categories of mild, moderate, and vigorous in a typical week. Time of vigorous activity was multiplied by 2 and added to moderate intensity exercise minutes. The GLTEQ has demonstrated convergent validity compared to accelerometer-based activity measures in cancer survivors [29]. In addition, participants were categorized as meeting or not meeting the PA guideline (≥150 or <150 minutes of MVPA per week) according to their self-reports.

#### Secondary Outcomes

Exercise capacity was measured by the 6-minute walk test (6MWT), which is a cost-effective measure that is well correlated with peak oxygen uptake (the gold standard measure) and is highly reproducible. The 6MWT was implemented according to American Thoracic Society guidelines [30]. Participants were instructed to walk at their fastest pace and cover the longest possible distance in 6 minutes.

Cancer-specific QoL was measured using the 30-item European Organisation for Research and Treatment of Cancer Quality of Life Questionnaire Core 30 (EORTC QLQ–C30) [31]. It covers 5 functional scales (physical, role, emotional, cognitive, and social), 9 symptom scales or items (fatigue, nausea and vomiting, pain, dyspnea, insomnia, appetite loss, constipation, diarrhea, and financial difficulties), and a global QoL scale. The global QOL score is analyzed in this study, with a higher score representing better QoL.

The M-PAC constructs were administered using a composite questionnaire [32] developed based on previous literature [33-41]. It covers 8 domains that were key constructs of reflective, regulatory, and reflexive processes that M-PAC was designed to facilitate. The constructs included affective attitude toward PA (3 items), instrumental attitude toward PA (3 items), perceived capability for PA (3 items), perceived opportunity for PA (3 items), decisional intentions to be physically active (1 item), behavioral regulation for PA (6 items), PA habit (4 items), and PA identity (4 items).

Demographic characteristics and disease information were collected using an investigator-designed background questionnaire at T0.

### Data Analysis

All statistical analyses were conducted in SPSS statistical software (version 28; IBM Corp). A 5% level of significance was used. Between-group differences in background characteristics and frequency of adverse events were assessed by a chi-square test or an independent *t* test as appropriate. App usage was summarized using descriptive analysis. To assess intervention effectiveness, 2 sets of analyses were performed. First, an intention-to-treat analysis was performed as the primary analysis. A generalized linear mixed-effects model, a statistical method that can accommodate missing data and does not require imputation of missing observations, was used [42]. Missing baseline Actigraph data due to noncompliance with wearing were imputed using multiple imputation. The group allocation was set as a fixed factor, and the intercept as a random factor. The Bonferroni adjustment was used for multiple pairwise comparisons. The change in continuous outcomes was compared using an identity link. The proportion of participants meeting the PA guideline (≥150 minutes of moderate-to-vigorous aerobic activity per week) is a dichotomous outcome variable, so a logit link was used. The group × time interaction was included in the model to test whether between-group differences varied over time. Potential covariates (ie, age, gender, cancer type, and time since last treatment) were included as fixed factors. Effect sizes of between-group change in continuous variables were computed in Cohen *d*. Second, per-protocol analysis was performed to investigate the influence of data from noncompliant participants and dropouts, including only participants who completed at least 75% of lessons and remained for the follow-up data collection.

### Ethical Considerations

Ethical approval was obtained from the Institutional Review Board of The University of Hong Kong/Hospital Authority Hong Kong West Cluster on August 18, 2021 (HKU/HA HKW IRB: UW 21‐485). All participants provided written informed consent before their involvement in the study. Following data collection, all study data were deidentified to ensure confidentiality. Each participant received a US $13 grocery market coupon as compensation for their participation.

## Results

### Participant Characteristics

**Figure 1.**
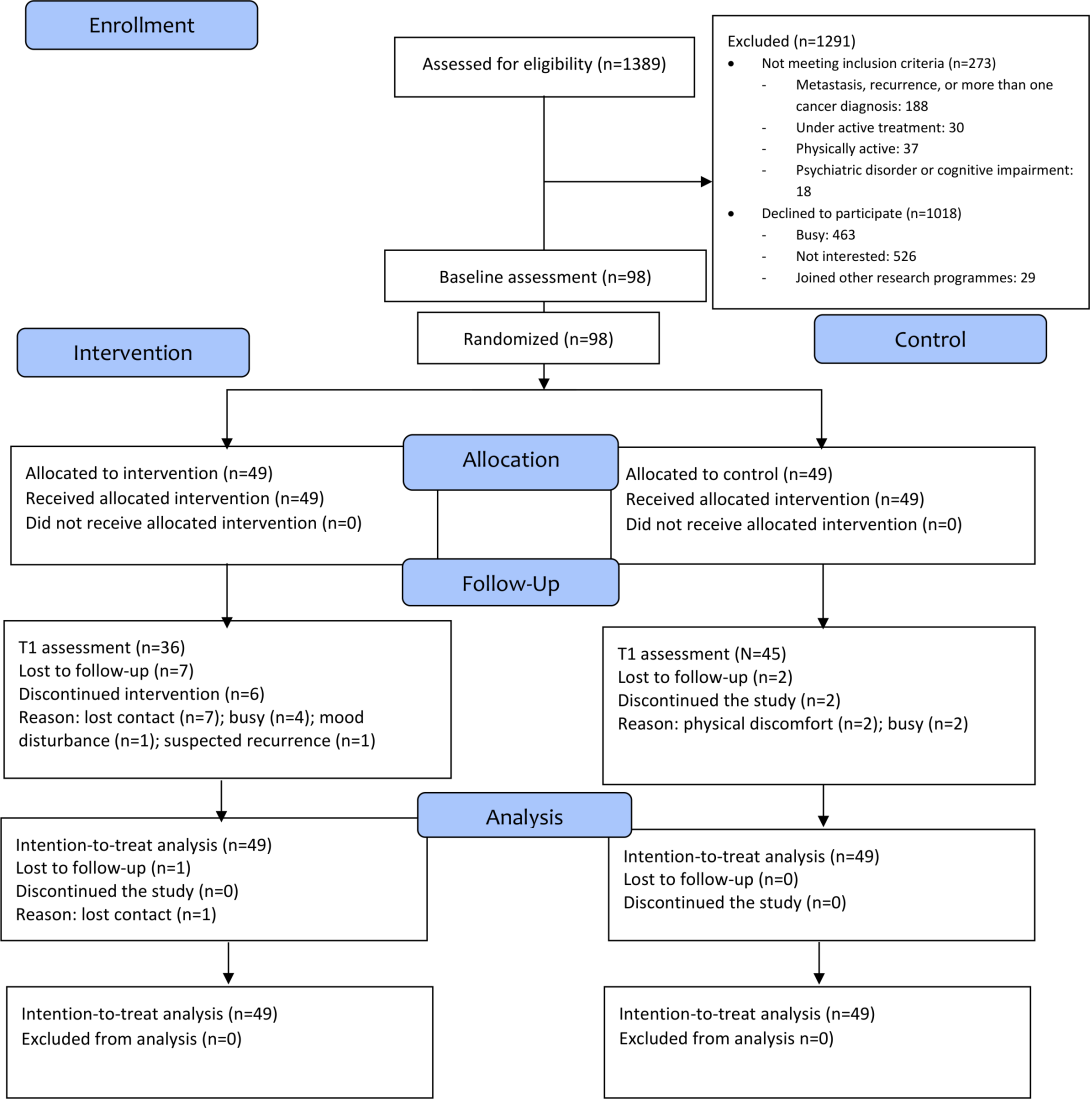
CONSORT (Consolidated Standards of Reporting Trials) flow diagram.

A total of 98 participants were recruited into the study, of whom 81 (82.7%) completed the study (36 in the intervention group and 45 in the control group; Figure 1). The main reason for dropout was a lack of time. The mean age of the sample was 54.54 (SD 7.58 years; Table 1). Of the participants, 85 (87%) were female, and 90 (92%) had received secondary education or above. Around two-thirds of participants were diagnosed with breast cancer (65/85, 66%) and early-stage cancer (53/98, 54%). The average number of months since cancer treatment completion was 78.62 (SD 60.18) months. A comparison of baseline characteristics between dropouts and completers is shown in Multimedia Appendix 1, which indicates no significant difference. For ActiGraph, the compliance rates were 78%, 78%, and 81% at T0, T1, and T2, respectively.

**Table 1.** Baseline characteristics.

Background characteristic	Overall	Intervention group (n=49)	Control group (n=49)	*P* value
Age (years), mean (SD)	54.54 (7.58)	53.67 (8.75)	55.41 (6.16)	.26
Sex, n (%)				.37
Male	13 (13.3)	8 (16.3)	5 (10.2)	
Female	85 (86.7)	41 (83.7)	44 (89.8)	
Education, n (%)				.46
Primary or below	8 (8.2)	3 (6.1)	5 (10.2)	
Secondary or above	90 (91.8)	46 (93.9)	44 (89.8)	
Type of cancer, n (%)				.29
Breast	65 (66.3)	30 (61.2)	35 (71.4)	
Other^a^	33 (33.7)	19 (38.8)	14 (28.6)	
Stage of cancer, n (%)				.48
Stage 0 and 1	53 (54.1)	23 (46.9)	30 (61.2)	
Stage 2 and 3	24 (24.5)	15 (30.6)	9 (18.4)	
Stage 4	1 (1.0)	0 (0.0)	1 (2.0)	
Completed chemotherapy, n (%)	41 (41.8)	21 (42.9)	20 (40.8)	.84
Time since treatment completion (months), mean (SD)	78.62 (60.18)	79.65 (66.72)	77.59 (53.52)	.89
Time since cancer diagnosis (months), mean (SD)	85.52 (59.53)	84.49 (66.64)	86.55 (52.13)	.87

a Other cancers include colorectal, lung, prostate, liver, stomach, thyroid, laryngeal, cervical, lymphoma, esophageal, bladder, and kidney.

Multimedia Appendix 2 shows the app usage statistics among completers in the intervention group. More than two-thirds of participants completed at least 75% of lessons (n=25). The number of participants completing classes decreased from the first (94.3%) to the last lesson (57.1%). No adverse events were reported.

### Intervention Effectiveness

#### Primary Outcome

For ActiGraph-measured MVPA, the group × time interaction was not significant (*P*=.74). There was no significant between-group difference in change at T1 (–8.54 minutes, 95% CI –36.19 to 19.11) and T2 (2.56 minutes, 95% CI –27.29 to 32.41; Table 2).

**Table 2.** Change in outcomes.

Outcome and time point	Intervention group (n=49), mean (95% CI)	Control group (n=49), mean (95% CI)	Intervention group change from baseline, mean (95% CI)	Control group change from baseline, mean (95% CI)	Between-group difference in change (intervention-control), mean (95% CI)	Effect size (Cohen d), mean (95% CI)	Group × time interaction, *P* value
ActiGraph MVPA^a^							.74
Baseline	52.33 (24.18 to 80.49)	45.07 (17.42 to 72.73)	—^b^	—	—	—	—
11 weeks (T1)	41.21 (11.59 to 70.82)	42.49 (14.06 to 70.92)	–11.12 (–35.91 to14.38)	–2.58 (–25.87 to 20.70)	–8.54 (–36.19 to 19.11)	–0.12 (–0.52 to 0.27)	—
23 weeks (T2)	49.41 (17.70 to 81.13)	39.59 (9.83 to 69.36)	–2.92 (–30.15 to 24.32)	–5.48 (–30.14 to 19.17)	2.56 (–27.29 to 32.41)	0.04 (–0.36 to 0.43)	—
Self-reported MVPA							.003
Baseline	16.61 (–6.04 to 39.26)	28.84 (6.64 to 51.04)	—	—	—	—	—
11 weeks (T1)	123.56 (78.43 to 168.69)	46.77 (5.18 to 88.36)	106.95 (57.67 to 156.22)	17.93 (–26.78 to 62.64)	89.02 (34.87 to 143.16)	0.66 (0.25 to 1.07)	—
23 weeks (T2)	82.95 (47.84 to 118.05)	45.81 (13.47 to 78.15)	66.34 (29.28 to 103.39)	16.97 (–16.70 to 50.64)	49.37 (8.63 to 90.10)	0.49 (0.09 to 0.89)	—
6MWT^c^ total walking distance							.05
Baseline	497.22 (460.59 to 533.86)	501.26 (465.30 to 537.21)	—	—	—	—	—
11 weeks (T1)	540.00 (499.19 to 580.81)	505.41 (466.54 to 544.27)	42.78 (14.49 to 71.06)	4.15 (–21.78 to 30.08)	38.63 (7.40 to 69.86)	0.50 (0.10 to 0.90)	—
23 weeks (T2)	542.29 (500.35 to 584.23)	520.90 (481.07 to 560.73)	45.07 (12.94 to 77.20)	19.65 (–9.97 to 49.26)	25.42 (–10.13 to 60.98)	0.29 (–0.11 to 0.69)	—
Global health status/QoL^d^ score							.80
Baseline	63.11 (56.17 to 70.04)	64.02 (57.21 to 70.82)	—	—	—	—	—
11 weeks (T1)	64.62 (57.25 to 71.98)	66.91 (59.97 to 73.85)	1.51 (–4.18 to 7.20)	2.89 (–2.37 to 8.15)	–1.38 (–7.69 to 4.93)	–0.09 (–0.48 to 0.31)	—
23 weeks (T2)	63.51 (55.67 to 71.35)	66.90 (59.55 to 74.25)	0.41 (–6.28 to 7.09)	2.88 (–3.24 to 9.01)	–2.48 (–9.85 to 4.90)	–0.14 (–0.53 to 0.26)	—

a MVPA: moderate-to-vigorous physical activity.

b Not applicable.

c 6MWT: 6-minute walk test.

d QoL: quality of life.

For self-reported MVPA, the group × time interaction was significant (*P*=.003), indicating a significant between-group difference in change over time. The intervention group showed a larger increase compared to the control group at both T1 (89.02 minutes, 95% CI 34.87-143.16) and T2 (49.37 minutes, 95% CI 8.63-90.10; Table 2). The proportion of participants meeting the 150 minutes per week MVPA guideline was significantly higher in the intervention group than in the control group at both T1 (27% vs 6.7%; AOR 5.09, 95% CI 1.25-20.78) and T2 (25.7% vs 4.7%; AOR 6.61, 95% CI 1.28-34.06).

#### Secondary Outcomes

For 6MWT, the group × time interaction was significant (*P*=.05). Specifically, the intervention group had a larger increase than the control group at T1 (25.42 meters, 95% CI –10.13 to 60.98). For QoL, neither group showed a significant change, and the group × time interaction was nonsignificant (Table 2).

For all M-PAC constructs, the group × time interaction was nonsignificant (Table 3). Of note, the intervention group showed significant improvement in affective attitude at T1 and T2, behavioral regulation at T1 and T2, habit at T1 and T2, and identity at T1 and T2, whereas the control group showed significant improvement in behavioral regulation at T1 and T2 and identity at T1 and T2.

**Table 3.** Change in multi-process action control (M-PAC) constructs.

Time point	Intervention group (n=49), mean (95% CI)	Control group (n=49), mean (95% CI)	Intervention group change from baseline, mean (95% CI)	Control group change from baseline, mean (95% CI)	Between-group difference in change (intervention-control), mean (95% CI)	Effect size (Cohen *d*), mean (95% CI)	Group × time interaction, *P* value
Affective attitude toward PA^a^							.24
Baseline	10.56 (9.55 to 11.56)	10.20 (9.21 to 11.18)	—^b^	—	—	—	—
11 weeks (T1)	11.53 (10.36 to 12.70)	10.38 (9.28 to 11.49)	0.97 (0.05 to 1.89)	0.19 (–0.65 to 1.03)	0.79 (–0.23 to 1.80)	0.31 (–0.09 to 0.71)	—
23 weeks (T2)	12.08 (10.94 to 13.23)	10.87 (9.80 to 11.95)	1.53 (0.56 to 2.49)	0.68 (–0.21 to 1.56)	0.85 (–0.21 to 1.91)	0.32 (–0.08 to 0.72)	—
Instrumental attitude toward PA							.26
Baseline	12.23 (11.53 to 12.93)	12.03 (11.34 to 12.71)	—	—	—	—	—
11 weeks (T1)	12.15 (11.33 to 12.96)	12.28 (11.53 to 13.04)	–0.08 (–0.81 to 0.65)	0.26 (–0.41 to 0.93)	–0.34 (–1.14 to 0.47)	–0.17 (–0.56 to 0.23)	—
23 weeks (T2)	12.56 (11.77 to 13.34)	12.09 (11.36 to 12.82)	0.33 (–0.40 to 1.06)	0.06 (–0.61 to 0.73)	0.27 (–0.54 to 1.08)	0.13 (–0.26 to 0.53)	—
Perceived capability over PA							.63
Baseline	11.07 (9.68 to 12.46)	11.29 (9.91 to 12.68)	—	—	—	—	—
11 weeks (T1)	11.48 (10.65 to 12.31)	11.01 (10.25 to 11.78)	0.41 (–1.12 to 1.94)	–0.28 (–1.79 to 1.23)	0.69 (–1.06 to 2.45)	0.16 (–0.24 to 0.55)	—
23 weeks (T2)	11.58 (10.66 to 12.51)	11.35 (10.50 to 12.20)	0.52 (–1.10 to 2.13)	0.06 (–1.52 to 1.64)	0.46 (–1.38 to 2.30)	0.10 (–0.30 to 0.50)	—
Perceived opportunity for PA							.75
Baseline	11.53 (10.87 to 12.20)	11.23 (10.57 to 11.88)	—	—	—	—	—
11 weeks (T1)	11.91 (11.11 to 12.72)	11.53 (10.78 to 12.28)	0.38 (–0.41 to 1.17)	0.31 (–0.42 to 1.03)	0.08 (–0.80 to 0.95)	0.03 (–0.36 to 0.43)	—
23 weeks (T2)	11.97 (11.20 to 12.75)	11.87 (11.15 to 12.59)	0.44 (–0.35 to 1.23)	0.65 (–0.09 to 1.38)	–0.21 (–1.08 to 0.67)	–0.10 (–0.49 to 0.30)	—
Behavioral regulation for PA							.49
Baseline	13.57 (11.12 to 16.03)	12.97 (10.56 to 15.37)	—	—	—	—	—
11 weeks (T1)	19.70 (16.99 to 22.41)	17.81 (15.27 to 20.34)	6.12 (3.78 to 8.47)	4.84 (2.67 to 7.01)	1.29 (–1.31 to 3.89)	0.20 (–0.20 to 0.59)	—
23 weeks (T2)	20.39 (17.47 to 23.31)	18.13 (15.40 to 20.86)	6.82 (4.25 to 9.38)	5.16 (2.82 to 7.50)	1.66 (–1.17 to 4.48)	0.24 (–0.16 to 0.63)	—
Habit of PA							.10
Baseline	13.90 (12.24 to 15.56)	13.41 (11.78 to 15.05)	—	—	—	—	—
11 weeks (T1)	15.72 (13.85 to 17.59)	14.44 (12.69 to 16.19)	1.82 (0.15 to 3.49)	1.03 (–0.52 to 2.58)	0.79 (–1.06 to 2.65)	0.17 (–0.23 to 0.57)	—
23 weeks (T2)	16.32 (14.53 to 18.11)	14.13 (12.45 to 15.82)	2.42 (0.99 to 3.85)	0.72 (–0.60 to 2.04)	1.70 (0.12 to 3.28)	0.43 (0.03 to 0.83)	—
PA identity							.80
Baseline	10.90 (9.65 to 12.15)	10.80 (9.58 to 12.03)	—	—	—	—	—
11 weeks (T1)	12.55 (11.05 to 14.04)	12.09 (10.68 to 13.50)	1.65 (0.44 to 2.85)	1.29 (0.19 to 2.38)	0.36 (–0.97 to 1.68)	0.11 (–0.29 to 0.51)	—
23 weeks (T2)	12.58 (11.11 to 14.04)	12.03 (10.65 to 13.41)	1.67 (0.45 to 2.90)	1.23 (0.11 to 2.35)	0.45 (–0.91 to 1.80)	0.13 (–0.27 to 0.53)	—
Decisional intentions to be physically active							.64
Baseline	6.66 (5.79 to 7.53)	6.50 (5.65 to 7.35)	—	—	—	—	—
11 weeks (T1)	7.17 (6.10 to 8.24)	6.71 (5.73 to 7.70)	0.51 (–0.60 to 1.61)	0.21 (–0.81 to 1.24)	0.29 (–0.93 to 1.51)	0.10 (–0.30 to 0.50)	—
23 weeks (T2)	6.95 (5.86 to 8.03)	6.15 (5.15 to 7.15)	0.29 (–0.91 to 1.48)	–0.35 (–1.47 to 0.76)	0.64 (–0.69 to 1.97)	0.19 (–0.21 to 0.59)	—

a PA: physical activity.

b Not applicable.

Per-protocol analysis was performed on the data of intervention group participants who had high compliance and completed all measurements (n=25), as well as control group participants who completed all measurements (n=45). The baseline characteristics were comparable between the 2 study groups (Multimedia Appendix 3). The results of the per-protocol analysis and the intention-to-treat analysis were similar (MultimediaAppendices 4  5).

## Discussion

### Principal Findings

To our knowledge, this is the first study to examine the effectiveness of a smartphone app, iteratively developed based on the M-PAC framework, WExercise, to promote PA among cancer survivors. For PA, mixed findings were identified, with self-reported MVPA showing a significant effect but ActiGraph-measured MVPA showing a nonsignificant effect. The app was also effective for increasing cancer survivors’ exercise capacity, but not their QoL. In terms of M-PAC constructs, the intervention group reported significant improvement in affective attitude toward PA, behavioral regulation for PA, habit of PA, and PA identity, while the control group improved only in behavioral regulation and PA identity; however, the change in between-group difference over time did not reach statistical significance.

Our study supported a significant effect on self-reported PA but not objectively measured PA. This discrepancy may be explained in several ways. First, the improvement in self-reported PA may be an overestimation caused by social desirability response bias. Second, activities of higher intensities are easier to recall due to feelings of tiredness, increasing the tendency to overreport. It is also possible that participants misinterpreted light-intensity activities as MVPA. Third, participants may have engaged in activities involving no vertical acceleration, such as cycling and upper-body movement, which might not be recorded efficiently using an ActiGraph [43]. Fourth, cancer survivors, a cohort with lower fitness, are more likely to report more MVPA but be measured as having less MVPA [44]. Fifth, compliance with ActiGraph wearing protocols may have affected the validity of ActiGraph data. In our study, 70%‐80% of participants complied with the wearing at each time point, which appears comparable to individuals with no cancer [45] and cancer populations [46]. The reasons for noncompliance were mainly discomfort, inconvenience, and allergic reactions, which align with the factors commonly reported in cancer survivors [47]. Although discrepancies in findings between objective and self-reported PA measurements have been consistently shown in the previous literature [48,49], the complementary use of both measures whenever possible is recommended. For our study, whether intervention effects are overstated by self-report or understated by ActiGraph is unclear. Although objectively measured PA is regarded as the gold standard of PA, the improvement in self-reported PA should not be ignored, given the significant improvement in cardiovascular fitness of the participants (ie, 6MWT), which is also an objective measure of physical well-being.

The M-PAC framework is a behavior change framework emphasizing action control, which differs from traditional behavior change theories that focus more on building intention rather than translating it into behavioral enactment [10]. Three pilot RCTs adopted the M-PAC framework to promote PA: 2 through website interventions for general adults [19,50] and another via telephone counseling for hematologic cancer survivors [20], with all showing promising results in MVPA improvement. Specifically, the telephone intervention for cancer survivors demonstrated a larger between-group change in self-reported MVPA [20] than our study, which may be explained by the higher intensity of interpersonal interactions in telephone counseling, resulting in a greater PA promotion effect than using technology alone [51]. Further research could explore the potential of combining interpersonal PA counseling with the WExercise app. Of note, our postintervention effect size still exceeds the small-to-moderate pooled effect size in previous meta-analyses on eHealth and mobile health studies for PA promotion in cancer survivors (*d*=0.26‐0.61) [5,52] and general populations (*d*=0.19‐0.28) [53], as well as PA promotion interventions in general (median *d* across meta-analyses=0.21) [54]. The smaller effect in previous studies may stem from interventions primarily using wearable devices without addressing behavior change [5,52,53]. In addition, most interventions were not guided by theories, limiting their effectiveness [5,52,53]. Notably, while previous meta-analyses reported diminishing effects on MVPA from postintervention to short-term follow-up [53], our study demonstrated sustained moderate effect sizes for self-reported MVPA from T1 to T2, suggesting considerable long-term sustainability.

Our study also extends previous research by secondary outcome analyses examining the changes in the M-PAC constructs, providing clues to how the intervention worked. While the nonsignificant group × time interaction in the M-PAC constructs may be explained by a lack of power, the within-group changes are worthwhile to explore. Both groups demonstrated significant improvement in regulation and identity, which may be explained by the fact that participants in both groups learned to regulate their PA behavior (eg, scheduling PA) after knowing about the recommended exercise guidelines and categorizing themselves as exercisers by enrolling in our study. Improvement in affect and habit was only reported in the intervention group with nonsignificant but small-to-medium-sized effects, but not in the control group, which may be triggered by the content of the app related to strategies to enhance the pleasure and enjoyment of PA and the use of prompts and cues to enact and maintain PA behavior. The lack of change in the other 2 constructs, perceived capability and perceived opportunity, highlights the need to strengthen the intervention on these aspects to enhance the ability and access to exercise, such as by including options for attending in-person or remotely delivered exercise sessions in conjunction with the app.

Approximately 70% of participants in our study completed more than 75% of lessons via the app. This appears to be comparable to a previous mobile health feasibility study promoting health behavior change among childhood cancer survivors, in which 57% of participants completed all 10 app-based education modules [55]. Notably, our per-protocol analysis, taking participant engagement into account, showed similar results to the intention-to-treat approach. While our usability test demonstrated promising initial engagement [23], continuous app refinement (eg, inclusion of enhanced app features) is likely necessary to maximize user engagement.

### Implications

Health care providers could recommend the WExercise app to inactive or insufficiently active cancer survivors who have no contraindications to unsupervised exercise, given its benefit in promoting self-reported PA and exercise capacity. To address the identified areas of improvement regarding app features and user interactivity, future iterations of the application should incorporate advanced features such as personalized feedback and incentives. Other novel functionalities should also be brainstormed to further differentiate the app from generic fitness trackers and enhance user engagement. Future studies may explore the feasibility and effectiveness of adding a few sessions of in-person PA training to the app intervention to maximize cost-effectiveness, as well as using customized behavior change techniques in the app to account for individual differences to better promote behavior change. In addition, future research should systematically investigate the potential factors causing incongruence between self-reported and objectively measured PA and explore strategies to enhance compliance with ActiGraph wearing to improve accuracy (eg, regular check-ins and rewear for missing days). Alternative self-reported measures and activity monitors of MVPA with higher agreement could be considered for use in future trials.

### Strengths and Limitations

Our study demonstrates several strengths. First, the WExercise app is uniquely driven by the M-PAC framework, addressing the intention-to-behavior gap by focusing on both intention formation and actionable strategies for behavior change. Second, the app was developed through a rigorous iterative process to ensure usability before the main trial. However, this study also has several limitations. First, most participants were educated and female, and they were recruited from one study site, limiting the generalizability of the findings. Second, the lack of participant blinding may have affected participant behavior and outcome reporting, introducing a risk of bias. Third, self-selection bias may have affected the findings because participants who signed up for the study may already have been motivated to be physically active, potentially overestimating the effectiveness of the app. Fourth, participants were sent reminders for missing classes, which may differ from routine practice and impact adherence in real-world settings.

### Conclusions

WExercise, a smartphone app, demonstrated significant benefits in increasing self-reported PA and exercise capacity at postintervention and 12 weeks postintervention, but not in ActiGraph-measured PA, M-PAC constructs, or QoL among cancer survivors. The app may be useful to promote PA in this population.

## Supplementary material

10.2196/75839 Multimedia Appendix 1 Comparison of baseline characteristics between participants who completed the study and those who dropped out.

10.2196/75839 Multimedia Appendix 2 App usage statistics among completers.

10.2196/75839 Multimedia Appendix 3 Per-protocol analysis of baseline characteristics.

10.2196/75839 Multimedia Appendix 4 Per-protocol analysis of change in outcomes.

10.2196/75839 Multimedia Appendix 5 Per-protocol analysis of change in multi-process action control (M-PAC) constructs.

10.2196/75839 Checklist 1 CONSORT-EHEALTH checklist.

## Abbreviations

6MWT: 6-minute walk test
CONSORT: Consolidated Standards of Reporting Trials
EORTC QLQ–C30: 30-item European Organisation for Research and Treatment of Cancer Quality of Life Questionnaire Core 30
GLTEQ: Godin Leisure Time Exercise Questionnaire
M-PAC: multi-process action control
MVPA: moderate-to-vigorous physical activity
PA: physical activity
QoL: quality of life
RCT: randomized controlled trial
WHO: World Health Organization
