# Supplementary material for: A Smartphone App (WExercise) to Promote Physical Activity Among Cancer Survivors: Randomized Controlled Trial
Source: J Med Internet Res. 2025 Oct 3;27:e75839. doi: 10.2196/75839 (PMC12494186; doi:10.2196/75839)
Supplement: Multimedia Appendix 1 [file jmir-v27-e75839-s001.docx]

Appendix 1. Comparison of baseline characteristics between participants who completed the study and who dropped out

| Background Characteristics | Overall, Number (%) | Number (%) | | *p*-Value |
| --- | --- | --- | --- | --- |
|  |  | Completed (*n*=81) | Drop Out (*n*=17) |  |
| Age, mean ± SD, years | 54.54 ± 7.58 | 54.57 ± 7.59 | 54.41 ± 7.79 | 0.939 |
| Gender | ·· | ·· | ·· | 0.841 |
| Male | 13 (13.3%) | 11 (13.6%) | 2 (11.8%) | ·· |
| Female | 85 (86.7%) | 70 (86.4%) | 15 (88.2%) | ·· |
| Education | ·· | ·· | ·· | 0.706 |
| Primary or below | 8 (8.2%) | 7 (8.6%) | 1 (5.9%) | ·· |
| Secondary or above | 90 (91.8%) | 74 (91.4%) | 16 (94.1%) | ·· |
| Type of cancer | ·· | ·· | ·· | 0.876 |
| Breast | 65 (66.3%) | 54 (66.7%) | 11 (64.7%) | ·· |
| Other^a^ | 33 (33.7%) | 27 (33.3%) | 6 (35.3%) | ·· |
| Stage of cancer | ·· | ·· | ·· | 0.585 |
| Stage 0 & 1 | 53 (54.1%) | 41 (50.6%) | 12 (70.6%) | ·· |
| Stage 2 & 3 | 24 (24.5%) | 21 (26%) | 3 (17.7%) | ·· |
| Stage 4 | 1 (1.0%) | 1 (1.2%) | 0 (0.0%) | ·· |
| Completed chemotherapy | 41 (41.8%) | 36 (44.4%) | 5 (29.4%) | 0.253 |
| Time since treatment completion, mean ± SD, months | 78.62 ± 60.18 | 82.12 ± 62.20 | 61.94 ± 47.47 | 0.210 |
| Time since cancer diagnosis, mean ± SD, months | 85.52 ± 59.53 | 89.95 ± 61.06 | 64.41 ± 47.62 | 0.108 |

^a^ Other cancers include colorectum, lung, prostate, liver, stomach, thyroid, laryngeal, cervical, lymphoma, esophageal, bladder, and kidney.
