# Supplementary material for: A Smartphone App (WExercise) to Promote Physical Activity Among Cancer Survivors: Randomized Controlled Trial
Source: J Med Internet Res. 2025 Oct 3;27:e75839. doi: 10.2196/75839 (PMC12494186; doi:10.2196/75839)
Supplement: Multimedia Appendix 2 [file jmir-v27-e75839-s002.docx]

Appendix 2. App usage statistics among completers

| App usage parameters (*n*=35) | Mean ± SD, n (%) |
| --- | --- |
| Number of participants completing at least 75% of lessons | 25 (69.44%) |
| Number of participants completing each online lesson  Introduction  Safety precaution  Week 1  Week 2  Week 3  Week 4  Week 5  Week 6  Week 7  Week 8  Week 9  Week 10 | 33 (94.3%)  32 (91.4%)  30 (85.7%)  31 (88.6%)  29 (82.9%)  28 (80%)  27 (77.1%)  24 (68.6%)  24 (68.6%)  23 (65.7%)  21 (60%)  20 (57.1%) |
| Mean number of online lessons completed (out of 12) | 9.33 ± 3.67 |
| Mean number of in-class quizzes completed (out of 4) | 2.74 ± 1.40 |
| Mean number of end-of-class quizzes completed (out of 10) | 6.69 ± 3.55 |
| Integrating step count | 327 (77.1%) |
| Logging PA | 22 (62.9%) |
