# Supplementary material for: A Smartphone App (WExercise) to Promote Physical Activity Among Cancer Survivors: Randomized Controlled Trial
Source: J Med Internet Res. 2025 Oct 3;27:e75839. doi: 10.2196/75839 (PMC12494186; doi:10.2196/75839)
Supplement: Multimedia Appendix 3 [file jmir-v27-e75839-s003.docx]

Appendix 3. Per-protocol analysis of baseline characteristics

| Background Characteristics | Overall, Number (%) | Group, Number (%) | | *p*-Value |
| --- | --- | --- | --- | --- |
|  |  | Intervention Group (*n*=25) | Control Group (*n*=45) |  |
| Age, mean ± SD, years | 54.93 ± 7.60 | 54.16 ± 9.75 | 55.36 ± 6.19 | 0.532 |
| Gender | ·· | ·· | ·· | 0.309 |
| Male | 10 (14.3%) | 5 (20.0%) | 5 (11.1%) | ·· |
| Female | 60 (85.7%) | 20 (80.0%) | 40 (88.9%) | ·· |
| Education | ·· | ·· | ·· | 0.309 |
| Primary or below | 6 (8.6%) | 1 (4.0%) | 5 (11.1%) | ·· |
| Secondary or above | 64 (91.4%) | 24 (96.0%) | 40 (88.9%) | ·· |
| Type of cancer | ·· | ·· | ·· | 0.110 |
| Breast | 45 (64.3%) | 13 (52.0%) | 32 (71.1%) | ·· |
| Other^a^ | 25 (35.7%) | 12 (48.0%) | 13 (28.9%) | ·· |
| Stage of cancer | ·· | ·· | ·· | 0.690 |
| Stage 0 & 1 | 36 (51.4%) | 10 (40.0%) | 26 (57.8%) | ·· |
| Stage 2 & 3 | 16 (22.8%) | 7 (28.0%) | 9 (20.0%) | ·· |
| Stage 4 | 1 (1.4%) | 0 (0.0%) | 1 (2.2%) | ·· |
| Completed chemotherapy | 30 (42.9%) | 11 (44.0%) | 19 (42.2%) | 0.885 |
| Time since treatment completion, mean ± SD, months | 81.91 ± 62.30 | 85.88 ±74.84 | 79.71 ± 54.93 | 0.694 |
| Time since cancer diagnosis, mean ± SD, months | 90.27 ± 61.18 | 90.16 ±75.42 | 90.33 ± 52.60 | 0.991 |

^a^ Other cancers include colorectum, lung, prostate, liver, stomach, thyroid, laryngeal, cervical, lymphoma, esophageal, bladder, and kidney.
