# Supplementary material for: A Smartphone App (WExercise) to Promote Physical Activity Among Cancer Survivors: Randomized Controlled Trial
Source: J Med Internet Res. 2025 Oct 3;27:e75839. doi: 10.2196/75839 (PMC12494186; doi:10.2196/75839)
Supplement: Multimedia Appendix 4 [file jmir-v27-e75839-s004.docx]

Appendix 4. Per-protocol analysis of change in outcomes

| Time Point | Intervention Group (*n*=25) | Control Group  (*n*=45) | Intervention Group Change from Baseline | | Control Group Change from Baseline | | Between-Group Difference in Change (Intervention-Control)  Effect Size | | | Time *p*-Value | Group *p*-Value | Group×Time Interaction *p*-Value |
| --- | --- | --- | --- | --- | --- | --- | --- | --- | --- | --- | --- | --- |
|  | Mean  (95% CI) | Mean  (95% CI) | Mean  (95% CI) | *p*-Value | Mean  (95% CI) | *p*-Value | Mean  (95% CI) | *p*-Value | Effect Size (Cohen’s d) (95%CI) | ·· | ·· | ·· |
| Actigraph MVPA | | | | | | | | | | | | |
| Baseline | 52.35 (16.14, 88.57) | 50.13 (19.88, 80.38) | – | – | – | – | – | – | – | 0.425 | 0.839 | 0.418 |
| 11 weeks (T1) | 33.07 (–2.22, 68.35) | 48.23 (17.84, 78.62) | –19.29 (–51.11, 12.54) | 0.423 | –1.90 (–26.06, 22.26) | 1.000 | –17.39 (–49.82, 15.04) | 0.288 | –0.22 (–0.61, 0.18) | – | – | – |
| 23 weeks (T2) | 49.76 (12.14, 87.38) | 44.87 (12.60, 77.13) | –2.59 (–35.08, 29.90) | 1.000 | –5.26 (–31.12, 20.61) | 1.000 | 2.67 (–31.07, 36.40) | 0.875 | 0.03 (–0.36, 0.43) | – | – | – |
| Self-reported MVPA | | | | | | | | | | | | |
| Baseline | 10.70 (–9.93, 31.32) | 32.29 (14.94, 49.65) | – | – | – | – | – | – | – | <0.001 | 0.018 | 0.001 |
| 11 weeks (T1) | 136.50 (85.56, 187.43) | 50.63 (11.86, 89.39) | 125.80 (64.54, 187.07) | <0.001 | 18.33 (–27.33, 64.00) | 0.984 | 107.47 (45.35, 169.58) | <0.001 | 0.70 (0.29, 1.11) | – | – | ·· |
| 23 weeks (T2) | 89.50 (52.65, 126.34) | 50.46 (21.48, 79.44) | 78.80 (34.04, 123.56) | <0.001 | 18.17 (–15.79, 52.12) | 0.580 | 60.63 (14.97, 106.30) | 0.010 | 0.54 (0.13, 0.94) | – | – | ·· |
| 6MWT total walking distance | | | | | | | | | | | | |
| Baseline | 486.36 (438.36, 534.36) | 497.10 (456.50, 537.69) | – | – | – | – | – | – |  | <0.001 | 0.312 | 0.014 |
| 11 weeks (T1) | 542.95 (490.56, 595.34) | 501.24 (457.79, 544.68) | 56.59 (20.68, 92.50) | <0.001 | 4.14 (–22.79, 31.07) | 1.000 | 52.45 (15.96, 88.93) | 0.005 | 0.58 (0.18, 0.98) | – | – | – |
| 23 weeks (T2) | 543.91 (490.92, 596.90) | 516.80 (472.65, 560.96) | 57.55 (17.91, 97.19) | 0.002 | 19.71 (–10.86, 50.28) | 0.354 | 37.84 (–2.84, 78.52) | 0.068 | 0.38 (–0.02, 0.78) | – | – | – |
| Global health status/QoL Score | | | | | | | | | | | | |
| Baseline | 61.30 (53.20, 69.41) | 62.05 (55.28, 68.82) | – | – | – | – | – | – | – | 0.751 | 0.513 | 0.871 |
| 11 weeks (T1) | 61.97 (53.65, 70.29) | 64.27 (57.35, 71.19) | 0.67 (–6.93, 8.27) | 1.000 | 2.22 (–3.44, 7.89) | 1.000 | –1.56 (–9.26, 6.15) | 0.688 | –0.08 (–0.48, 0.32) | – | – | – |
| 23 weeks (T2) | 61.30 (52.29, 70.31) | 64.24 (56.82, 71.66) | 0 (–8.53, 8.53) | 1.000 | 2.19 (–4.26, 8.65) | 1.000 | –2.19 (–10.89, 6.50) | 0.616 | –0.10 (–0.50, 0.30) | – | – | – |
