# Supplementary material for: A Smartphone App (WExercise) to Promote Physical Activity Among Cancer Survivors: Randomized Controlled Trial
Source: J Med Internet Res. 2025 Oct 3;27:e75839. doi: 10.2196/75839 (PMC12494186; doi:10.2196/75839)
Supplement: Multimedia Appendix 5 [file jmir-v27-e75839-s005.docx]

Appendix 5. Per-protocol analysis of change in MPAC constructs

| Time Point | Intervention Group (*n*=25) | Control Group (*n*=45) | Intervention Group Change from Baseline | | Control Group Change from Baseline | | Between-Group Difference in Change (Intervention-Control) | | | Time *p*-Value | Group *p*-Value | Group×Time Interaction *p*-Value |
| --- | --- | --- | --- | --- | --- | --- | --- | --- | --- | --- | --- | --- |
|  | Mean  (95% CI) | Mean  (95% CI) | Mean  (95% CI) | *p*-Value | Mean  (95% CI) | *p*-Value | Mean  (95% CI) | *p*-Value | Effect Size (Cohen’s d) (95%CI) | ·· | ·· | ·· |
| Affective attitude toward PA | | | | | | | | | | | | |
| Baseline | 10.60 (9.31, 11.89) | 10.08 (8.99, 11.16) | – | – | – | – | – | – | – | 0.004 | 0.053 | 0.384 |
| 11 weeks  (T1) | 11.48 (10.10, 12.95) | 10.23 (9.03, 11.43) | 0.88 (–0.30, 2.06) | 0.214 | 0.16 (–0.72, 1.04) | 1.000 | 0.72 (–0.47, 1.92) | 0.231 | 0.24 (–0.16, 0.64) | – | – | ·· |
| 23 weeks  (T2) | 12.04 (10.67, 13.41) | 10.71 (9.56, 11.85) | 1.44 (0.25. 2.63) | 0.013 | 0.63 (–0.27, 1.53) | 0.269 | 0.81 (–0.40, 2.02) | 0.188 | 0.27 (–0.13, 0.66) | – | – | ·· |
| Instrumental attitude toward PA | | | | | | | | | | | | |
| Baseline | 11.88 (10.94, 12.82) | 12.00 (11.21, 12.79) | – | – | – | – | – | – | – | 0.339 | 0.711 | 0.347 |
| 11 weeks  (T1) | 12.24 (11.19, 13.29) | 12.22 (11.36, 13.08) | 0.36 (–0.57, 1.29) | 1.000 | 0.22 (–0.47, 0.92) | 1.000 | 0.14 (–0.81, 1.08) | 0.772 | 0.06 (–0.34, 0.46) | – | – | – |
| 23 weeks  (T2) | 12.52 (11.52, 13.51) | 12.02 (11.19, 12.84) | 0.64 (–0.28, 1.56) | 0.278 | 0.02 (–0.68, 0.71) | 1.000 | 0.62 (–0.32, 1.56) | 0.191 | 0.27 (–0.13, 0.66) | – | – | – |
|  | Perceived capability over PA | | | | | | | | | | | |
| Baseline | 11.36 (9.19, 13.54) | 11.22 (9.55, 12.89) | – | – | – | – | – | – |  | 0.610 | 0.490 | 0.691 |
| 11 weeks  (T1) | 11.60 (10.60, 12.61) | 10.95 (10.10, 11.80) | 0.24 (–2.21, 2.69) | 1.000 | –0.27 (–2.09, 1.56) | 1.000 | 0.51 (–1.98, 2.99) | 0.685 | 0.08 (–0.31, 0.48) | – | – | – |
| 23 weeks  (T2) | 11.64 (10.54, 12.74) | 11.29 (10.38, 12.21) | 0.28 (–2.23, 2.79) | 1.000 | 0.07 (–1.80, 1.95) | 1.000 | 0.21 (–2.34, 2.75) | 0.872 | 0.03 (–0.36, 0.43) | – | – | – |
|  | Perceived opportunity for PA | | | | | | | | | | | |
| Baseline | 11.58 (10.76, 12.41) | 11.22 (10.53, 11.91) | – | – | – | – | – | – |  | 0.129 | 0.208 | 0.339 |
| 11 weeks  (T1) | 12.22 (11.24, 13.20) | 11.47 (10.67, 12.26) | 0.64 (–0.34, 1.62) | 0.335 | 0.24 (–0.48, 0.97) | 1.000 | 0.40 (–0.59, 1.38) | 0.427 | 0.16 (–0.23, 0.56) | – | – | – |
| 23 weeks  (T2) | 11.94 (11.03, 12.86) | 11.79 (11.04, 12.55) | 0.36 (–0.59, 1.31) | 1.000 | 0.57 (–0.14, 1.28) | 0.161 | –0.21 (–1.17, 0.75) | 0.664 | –0.09 (–0.48, 0.31) | – | – | – |
|  | Behavioral regulation for PA | | | | | | | | | | | |
| Baseline | 13.54 (10.36, 16.73) | 12.87 (10.19, 15.54) | – | – | – | – | – | – |  | <0.001 | 0.150 | 0.444 |
| 11 weeks  (T1) | 20.34 (16.98, 23.70) | 17.74 (14.95, 20.52) | 6.80 (3.80, 9.81) | <0.001 | 4.87 (2.63, 7.11) | <0.001 | 1.93 (–1.11, 4.98) | 0.210 | 0.26 (–0.14, 0.65) | – | – | – |
| 23 weeks  (T2) | 20.34 (16.71, 23.98) | 18.05 (15.06, 21.04) | 6.80 (3.67, 9.93) | <0.001 | 5.18 (2.82, 7.55) | <0.001 | 1.61 (–1.57, 4.81) | 0.316 | 0.20 (–0.19, 0.60) | – | – | – |
|  | Habit of PA | | | | | | | | | | | |
| Baseline | 13.16 (11.14, 15.18) | 13.30 (11.61, 15.00) | – | – | – | – | – | – |  | 0.002 | 0.191 | 0.106 |
| 11 weeks  (T1) | 15.64 (13.39, 17.89) | 14.23 (12.39, 16.08) | 2.48 (0.32, 4.64) | 0.019 | 0.93 (–0.68, 2.55) | 0.479 | 1.55 (–0.65, 3.74) | 0.164 | 0.28 (–0.11, 0.68) | – | – | – |
| 23 weeks  (T2) | 15.76 (13.58, 17.94) | 13.94 (12.13, 15.74) | 2.60 (0.80, 4.40) | 0.002 | 0.63 (–0.73, 1.99) | 0.769 | 1.97 (0.13, 3.80) | 0.036 | 0.43 (0.03, 0.83) | – | – | – |
|  | PA identity | | | | | | | | | | | |
| Baseline | 11.11 (9.43, 12.79) | 10.92 (9.50, 12.34) | – | – | – | – | – | – | – | <0.001 | 0.410 | 0.713 |
| 11 weeks  (T1) | 12.95 (11.02, 14.89) | 12.19 10.60, 13.78) | 1.84 (0.30, 3.38) | 0.013 | 1.27 (0.12, 2.41) | 0.025 | 0.57 (–0.98, 2.13) | 0.465 | 0.15 (–0.25, 0.55) | – | – | – |
| 23 weeks  (T2) | 12.91 (11.03, 14.80) | 12.19 (10.60, 13.68) | 1.80 (0.26, 3.34) | 0.017 | 1.20 (0.03, 2.36) | 0.042 | 0.60 (–0.97, 2.17) | 0.447 | 0.16 (–0.24, 0.55) | – | – | – |
|  | Decisional intentions to be physically active | | | | | | | | | | | |
| Baseline | 6.61 (5.45, 7.78) | 6.56 (5.59, 7.53) | – | – | – | – | – | – |  | 0.198 | 0.221 | 0.572 |
| 11 weeks  (T1) | 7.49 (6.15, 8.83) | 6.71 (5.93, 7.80) | 0.88 (–0.61, 2.37) | 0.455 | 0.16 (–0.96, 1.27) | 1.000 | 0.72 (–0.79, 2.24) | 0.342 | 0.19 (–0.20, 0.59) | – | – | – |
| 23 weeks  (T2) | 6.93 (5.60, 8.27) | 6.13 (5.04, 7.22) | 0.32 (–1.23, 1.87) | 1.000 | –0.43 (–1.60, 0.74) | 1.000 | 0.75 (–0.83, 2.33) | 0.349 | 0.19 (–0.21, 0.59) | – | – | – |
